# Predicting Grating Orientations With Cross-Frequency Coupling and Least Absolute Shrinkage and Selection Operator in V1 and V4 of Rhesus Monkeys

**DOI:** 10.3389/fncom.2020.605104

**Published:** 2021-01-25

**Authors:** Zhaohui Li, Yue Du, Youben Xiao, Liyong Yin

**Affiliations:** ^1^School of Information Science and Engineering, Yanshan University, Qinhuangdao, China; ^2^Hebei Key Laboratory of Information Transmission and Signal Processing, Yanshan University, Qinhuangdao, China; ^3^Department of Neurology, The First Hospital of Qinhuangdao, Qinhuangdao, China

**Keywords:** orientation selectivity, visual cortex, cross-frequency coupling, local field potential, LASSO

## Abstract

Orientation selectivity, as an emergent property of neurons in the visual cortex, is of critical importance in the processing of visual information. Characterizing the orientation selectivity based on neuronal firing activities or local field potentials (LFPs) is a hot topic of current research. In this paper, we used cross-frequency coupling and least absolute shrinkage and selection operator (LASSO) to predict the grating orientations in V1 and V4 of two rhesus monkeys. The experimental data were recorded by utilizing two chronically implanted multi-electrode arrays, which were placed, respectively, in V1 and V4 of two rhesus monkeys performing a selective visual attention task. The phase–amplitude coupling (PAC) and amplitude–amplitude coupling (AAC) were employed to characterize the cross-frequency coupling of LFPs under sinusoidal grating stimuli with different orientations. Then, a LASSO logistic regression model was constructed to predict the grating orientation based on the strength of PAC and AAC. Moreover, the cross-validation method was used to evaluate the performance of the model. It was found that the average accuracy of the prediction based on the combination of PAC and AAC was 73.9%, which was higher than the predicting accuracy with PAC or AAC separately. In conclusion, a LASSO logistic regression model was introduced in this study, which can predict the grating orientations with relatively high accuracy by using PAC and AAC together. Our results suggest that the principle behind the LASSO model is probably an alternative direction to explore the mechanism for generating orientation selectivity.

## Introduction

Orientation is a basic and important characteristic of natural images. The detection of oriented stimuli is generally known as orientation selectivity, i.e., neurons respond preferentially to elongated stimuli oriented along a specific axis in the visual field but respond weakly to stimuli oriented orthogonally to their preferred axis (Antinucci et al., 2016). Orientation selectivity was first observed in cat primary visual cortex nearly 60 years ago (Hubel and Wiesel, 1962). Since then, numerous studies investigated the orientation selectivity in visual systems of vertebrates and invertebrates, such as rodents (Niell and Stryker, 2008), primates (Hubel and Wiesel, 1968; Fisher et al., 2015), fish (Nikolaou et al., 2012), and insects (Fisher et al., 2015). Moreover, it plays a key role in shape perception and other visual information processing (Mansfield, 1974; Girshick et al., 2011; Lien and Scanziani, 2018; Crijns et al., 2019). However, as an emergent property of neurons in the visual cortex, how the orientation selectivity generates is still debated. In the past decades, many studies have been devoted to exploring the mechanism for generating the orientation selectivity. For example, by measuring the dynamics of orientation tuning of single neurons in the V1 cortex of macaque monkeys, it was found that orientation selectivity is generated mainly by both tuned enhancement and global suppression (Shapley et al., 2003); orientation selectivity is similar for lateral geniculate nucleus relay cells spiking and subthreshold input to V1 neurons, indicating that cortical orientation selectivity is inherited from the lateral geniculate nucleus in mouse (Scholl et al., 2013); both the preferred orientation and the width of orientation tuning were well-predicted by a feedforward model of orientation selectivity, which was constructed based on simple cells in the cat visual cortex (Lampl et al., 2001). Because the neuronal firings cannot reflect the synaptic activity of neurons in a local region, which is closely related to advanced neurological functions, the neural oscillations have also been applied to investigate orientation selectivity. For instance, it is possible to estimate the orientation selectivity of stimulus-evoked LFP signals in primary visual cortex on the basis of the surrounding map of orientation preference (Katzner et al., 2009); there is a weak correlation between the preferred orientation of multi-unit activity and gamma-band LFP recorded on the same tetrode, while there is a strong correlation between the ocular preferences of both signals (Berens, 2008). In this study, we also used some features extracted from neural oscillations to characterize the orientation selectivity.

In fact, neural oscillations are rhythmic patterns of electrical activity produced by the interaction of neurons in the nervous system (Buzsaki, 2004), which have been ubiquitously observed in the mammalian brain and involved in many brain functions (Fries, 2005; Womelsdorf et al., 2007; Minarik et al., 2018). An essential characteristic of neural oscillations is that they coordinate across spatial and temporal scales, which can be depicted by cross-frequency coupling (CFC) (Jensen and Colgin, 2007; Canolty and Knight, 2010). As well-known, while larger populations generally oscillate and synchronize at lower frequencies, smaller ensembles are active at higher frequencies (Buzsaki, 2006). Therefore, CFC would facilitate flexible coordination of neural activity simultaneously in time and space (Aru et al., 2015). It is not only the primary manner for the nervous system to encode external stimuli but also an important way to express and exchange information (Belluscio et al., 2012; Hyafil et al., 2015; Zheng et al., 2016; Zhang et al., 2017; Yeh and Shi, 2018). Based on the three properties of a signal, i.e., frequency, amplitude, and phase, there are four fundamental types of CFC, including phase–frequency coupling (PFC) (Roberts et al., 2013), phase–phase coupling (PPC) (Belluscio et al., 2012), phase–amplitude coupling (PAC) (Tort et al., 2010), and amplitude–amplitude coupling (AAC) (Yeh et al., 2016). PAC reflects the degree that the amplitude of higher-frequency oscillations is modulated by the phase of lower-frequency oscillations (Canolty et al., 2006), which is the most common and important type of CFC and plays a major role in the brain functions such as motion (Cheung et al., 2019; Khamechian and Daliri, 2020), memory (Tseng et al., 2019), learning (Zaleshin and Merzhanova, 2019), and sleep (Cox et al., 2019). There are many algorithms to estimate PAC, such as mean vector length (MVL) (Canolty et al., 2006), modulation index (MI) (Tort et al., 2008), and generalized eigendecomposition-based cross-frequency coupling framework (gedCFC) (Cohen, 2017). Among these, the gedCFC can effectively identify false couplings and weak patterns of CFC in noisy data accurately (Cohen, 2017). Thus, it was employed in this study to measure the PAC of LFP in V1 and V4. On the other hand, AAC measures the correlation between the amplitude envelopes of two neural oscillations at different frequencies (Yeh et al., 2016). It has also been used to explore some brain functions, e.g., the AAC between theta and low-frequency gamma (30–50 Hz) waves in the hippocampus can predict the spatial memory performance of rats (Shirvalkar et al., 2010). In this study, a method based on the Pearson correlation coefficient was used to investigate the AAC available in V1 and V4. In addition, the PFC and PPC were not observed to vary with the grating orientation. Thus, they were not discussed in the next sections.

Considering the wide application and good effects of CFC in studying brain functions, we constructed a LASSO logistic regression model based on PAC and AAC to predict the grating orientations. We hope it could provide an alternative direction to explore the mechanism of orientation selectivity. LASSO is a regression analysis method that performs both variable selection and regulation to enhance the prediction accuracy and interpretability of the statistical model it produces (Friedman et al., 2010; Ciuperca, 2012). It can effectively avoid the problems of over-fitting and high correlation in the least squares estimation, and solve the problem of multicolinearity in regression analysis (Zhou et al., 2017; Zhang et al., 2018). LASSO has been widely used in many fields, such as the prediction of disease outcomes (Tang et al., 2018) and genome-wide (Waldmann et al., 2019). Because of its uncertainty and randomness, the LASSO logistic regression model is very suitable to simulate the encoding process of the nervous system (Traub et al., 2004; Palva et al., 2005). Moreover, it has been proved that LASSO works well for any degree of correlation if suitable tuning parameters are chosen (Hebiri and Lederer, 2013). Therefore, in this study, we combined PAC and AAC to build a prediction model for the grating orientation based on the LASSO logistic regression model.

## Materials and Methods

### Experiment Procedure and Visual Stimulation

All procedures were conducted in compliance with the National Institutes of Health Guide for the Care and Use of Laboratory Animals, and were approved by the Institutional Animal Care and Use Committee of Beijing Normal University. Two adult male rhesus monkeys were used for data recording in the experiment. During behavioral training, a titanium post was attached to the skull with bone screws to immobilize the animal's head. The general anesthesia was induced with ketamine (10 mg/kg) and maintained with isoflurane (1.5–2.0%). The monkeys were first trained to accomplish a simple fixation task. Then, two 6 × 8 multi-electrode arrays (with electrode length 0.5–0.6 mm, interelectrode spacing 0.4 mm, and typical electrode impedance was a few hundred kiloohms; Blackrock Microsystems) were chronically implanted into V1 and V4, respectively (Li et al., 2016, 2019). The neural electrophysiological data were recorded at 10 kHz using a 128-channel Cerebus system (Blackrock Microsystems).

In the experiment, the visual stimuli were generated by a stimulus generator system (ViSaGe) and presented on a 22-inch CRT monitor with a viewing distance of 100 cm. The visual stimuli, i.e., drifting sinusoidal gratings, were displayed within a circular patch of 4° visual angle in diameter. Other parameters of the gratings were constant in the whole experiment, such as the temporal frequency of 4 Hz, the spatial frequency of 2 cycles/degree, and the contrast of 99%. Every stimulus was displayed on the screen for 2 s and repeated 30 times. On each trial, the grating appeared in a pseudorandom order with the orientation ranging from 0° to 360° in steps of 22.5°. After a lever was pulled by the animal, a fixation point (FP) of 0.1° was presented on the CRT center. Within the next 600 ms, the animal was required to maintain its fixation on the circular area of 0.6° in radius around the FP for 200 ms. The stimulus was displayed for 2 s, followed by a 200-ms blank interval. Then, the FP was slightly dimmed, and the animal must release the lever within 600 ms for a drop of juice as reward (Li et al., 2016, 2019).

### Phase–Amplitude Coupling

The generalized eigendecomposition-based cross-frequency coupling framework (gedCFC) was employed to measure the PAC in this study. The gedCFC combines source-separation algorithms and the dynamics of mesoscopic neurophysiological processes to conceptualize CFC as network interactions with diverse spatial or topographical distributions (Cohen, 2017). Eigendecomposition involves finding certain vectors that are associated with square matrices. The basic eigenvalue equation is *Ax* = λ*x*, where *A* is a square matrix, *x* is the eigenvector, and λ is the eigenvalue. It means that multiplying the eigenvector *x* by matrix *A* has the same outcome as multiplying *x* by a single number λ. In other words, matrix *A* merely stretches or shrinks *x* without changing its direction.

The eigenvalue equation can be generalized to two square matrices *A* and *B*, as *Ax* = *Bx*λ. If *A* is a covariance matrix of a “signal” dataset, and *B* is a covariance matrix of a “reference” dataset, then the generalized eigendecomposition can be understood to produce eigenvectors that identify directions of maximal power ratio in the matrix product *B*^−1^*A*, i.e., directions that best differentiate matrix *A* from *B*. The gedCFC identifies multichannel CFC-related networks by contrasting covariance matrices computed from to-be-maximized data features (matrix *A*) against to-be-minimized data features (matrix *B*). The two covariance matrices should be similar enough to suppress CFC-unrelated activity, while being different enough to isolate the neural networks that exhibit CFC. More details of the gedCFC algorithm can be found in the reference (Cohen, 2017).

### Amplitude–Amplitude Coupling

The Pearson correlation coefficient was used to measure the amplitude–amplitude coupling strength of LFPs. First is the band-pass filtering the LFPs in V1 and V4 to obtain low-frequency (4–12 Hz) and high-frequency (30–90 Hz) neural oscillations, denoted as *x*(*t*) and *y*(*t*), respectively. Second is the extracting of the amplitude of *x*(*t*) by:

(1) $$\begin{array}{l} A\left(t\right)=\sqrt{x{\left(t\right)}^{2}+H{\left[x\left(t\right)\right]}^{2}} \end{array}$$

where *H*[*x*(*t*)] is the Hilbert transform of *x*(*t*):

(2) $$\begin{array}{l} H\left[x\left(t\right)\right]=x\left(t\right)*\frac{1}{\pi t}=\frac{1}{\pi }\int_{-\infty }^{+\infty }\frac{x\left(\tau \right)}{t-\tau }d\tau . \end{array}$$

Similarly, we can get the amplitude *B*(*t*) of *y*(*t*). Finally, the Pearson correlation coefficient between *A*(*t*) and *B*(*t*) as the strength of AAC is calculated:

(3) $$\begin{array}{l} r=corr\left(A\left(t\right),B\left(t\right)\right)=\frac{cov\left(A\left(t\right),B\left(t\right)\right)}{\sigma \left(A\left(t\right)\right)\sigma \left(B\left(t\right)\right)}\;. \end{array}$$

### Least Absolute Shrinkage and Selection Operator Logistic Regression Model

For an ordinary linear model:

(4) $$\begin{array}{l} Y=X\beta +\varepsilon \end{array}$$

where $Y={\left(y_{1},y_{2},\ldots ,y_{n}\right)}^{T}$ is the response variable, *X* = (*X*^(1)^, *X*^(2)^, …, *X*^(*d*)^) is the covariate, $\beta ={\left(\beta_{1},\beta_{2},\ldots ,\beta_{d}\right)}^{T}$ is the regression coefficient, $\varepsilon ={\left(\varepsilon_{1},\varepsilon_{2},\ldots ,\varepsilon_{n}\right)}^{T}$ is the random variable and $\varepsilon_{i}\sim N\left(0,\sigma^{2}\right)$. The smallest penalty likelihood function is used as the regression coefficient estimate (Breiman, 1995; Tibshirani, 1996), which is calculated by

(5) $$\begin{array}{l} \overset{⌢}{\beta }=\arg\underset{\beta \in R^{d}}{\min}\left(||Y-X\beta |{|}^{2}+\lambda \sum_{j\;=\;1}^{d}|\beta_{j}|\right), \end{array}$$

where λ is the weight coefficient of LASSO.

**Figure 1:**
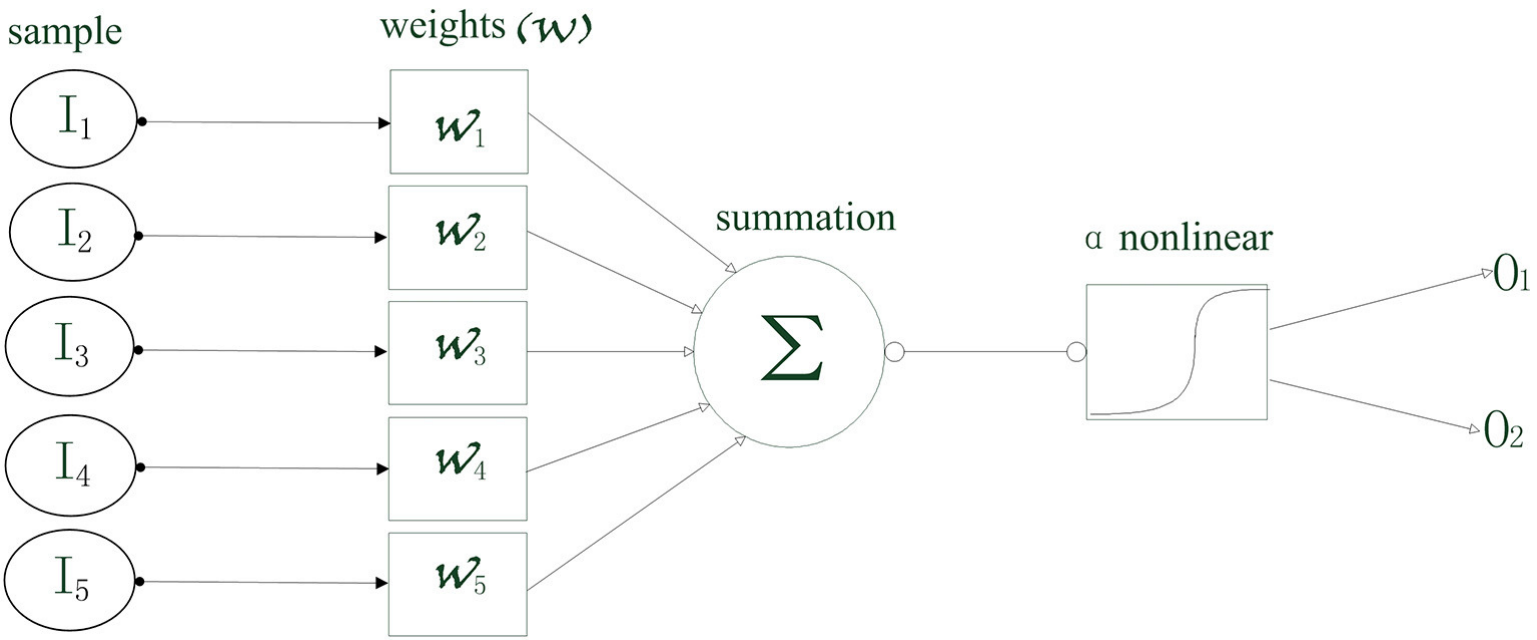
The schematic diagram of the least absolute shrinkage and selection operator (LASSO) logistic regression model.

In this study, the input data were the PAC and AAC, including the strength values in V1 and V4, respectively, and the strength values between V1 and V4. The output is the grating orientation corresponding to the cross-frequency coupling strength. If a specific input is denoted by *I*, then the conditional probability of the corresponding output (*O*_1_ and *O*_2_) can be calculated by:

(6) $$\begin{array}{l} P_{r}\left(O=O_{1}|I\right)=\frac{1}{1+e^{-x^{T}w}}\;, \end{array}$$

(7) $$\begin{array}{l} P_{r}\left(O=O_{2}|I\right)=\frac{1}{1+e^{x^{T}w}}\;. \end{array}$$

where *x* is the cross-frequency coupling strength, and *w* is the weight vector. As shown in Figure 1, the inputs are first multiplied by the weight vectors, respectively, and then added up. Next, a non-linear logic process is solved, i.e.,:

(8) $$\begin{array}{l} \partial \left(x\right)=\frac{\text{1}}{\left(1+e^{x}\right)}\;. \end{array}$$

Therefore, the conditional probabilities can be calculated, which represents the possibility that an output corresponded to the input. The weighted vector to the larger possibility is determined as the feature obtained by training the current data set. In this study, the weight of the penalty function is set to 100, and the penalty function takes the Elastic Net. Here, we briefly introduced the main idea of the LASSO model. The training and predicting were implemented by using the glmnet toolbox in MATLAB.

In order to test the performance of the model, the cross-validation method was employed in this study. The error of prediction and the sum of their squares were calculated (Hawkins et al., 2003; Braga-Neto and Dougherty, 2004; Vehtari et al., 2016). In each verification, all samples were randomly divided into M parts. M – 2 of them were used as training set, and the remaining 2 as testing sets. The correct rate for each prediction is denoted by *R*_*i*_, *i* = 1, 2, ⋯, *L*, where *L* is the total number of the training trials. Therefore, the overall accuracy of the prediction is calculated as:

(9) $$\begin{array}{l} \text{R}=\frac{\sum_{i\;=\;1}^{L}R_{i}}{L} \end{array}$$

## Results

**Figure 2:**
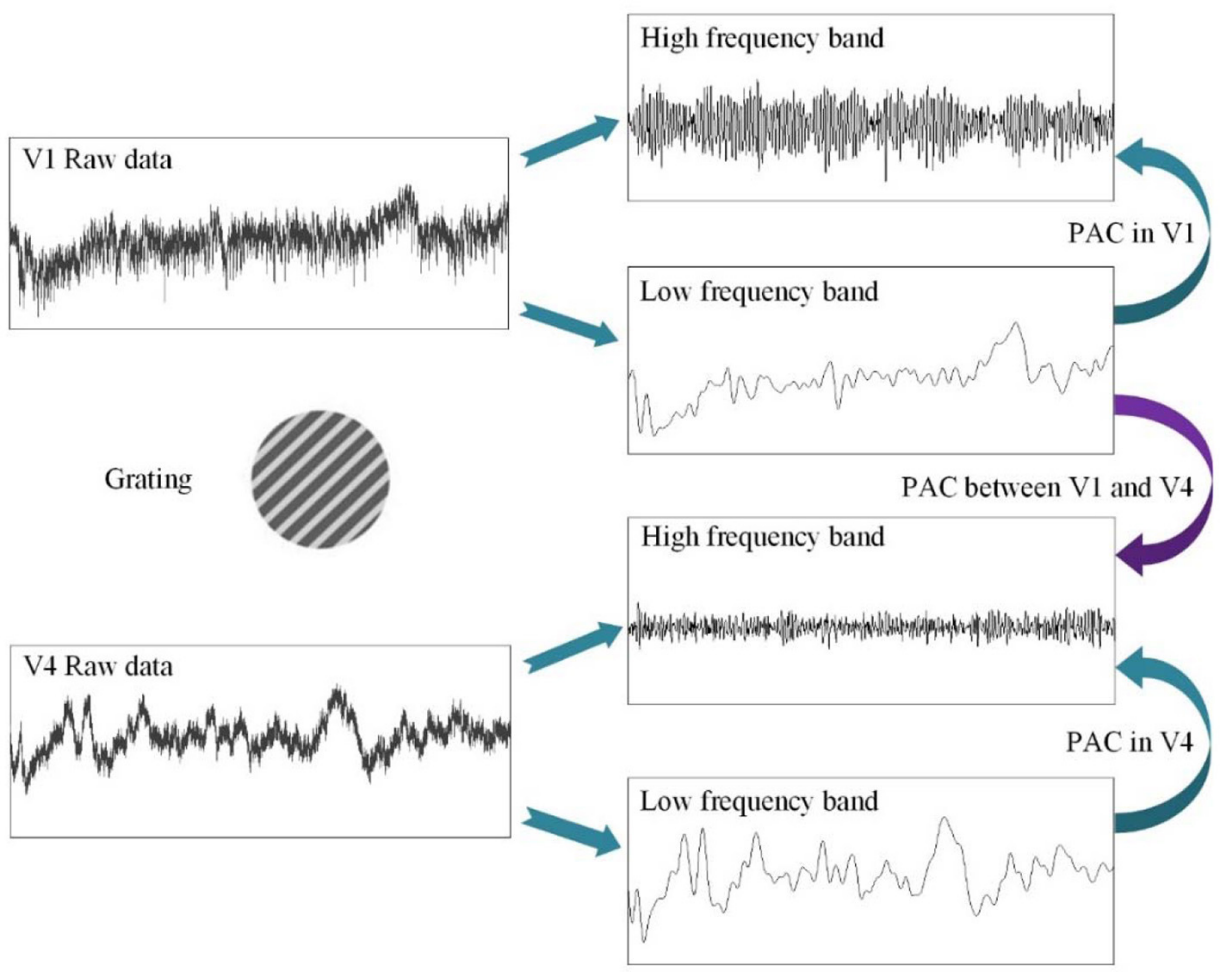
The schematic diagram for the calculating procedure of phase–amplitude coupling (PAC).

**Figure 3:**
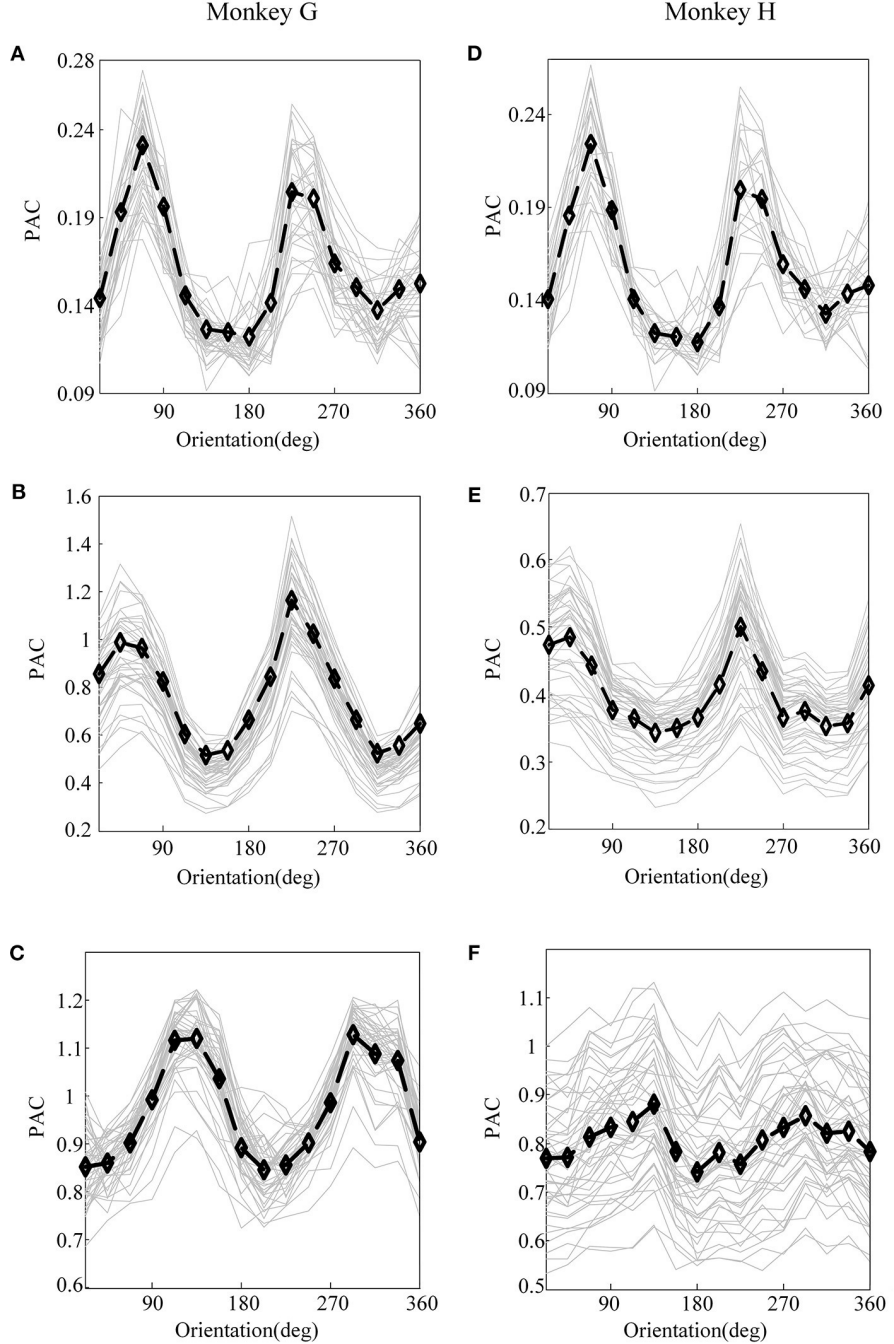
The PAC of local field potential (LFP) for monkey G and monkey H. **(A)** The PAC between V1 and V4 of monkey G. **(B)** The PAC in V1 of monkey G. **(C)** The PAC in V4 of monkey G. **(D)** The PAC between V1 and V4 of monkey H. **(E)** The PAC in V1 of monkey H. **(F)** The PAC in V4 of monkey H. In these panels, the gray lines represent the PAC of LFP recorded by individual electrodes, and the black lines plot their means.

**Figure 4:**
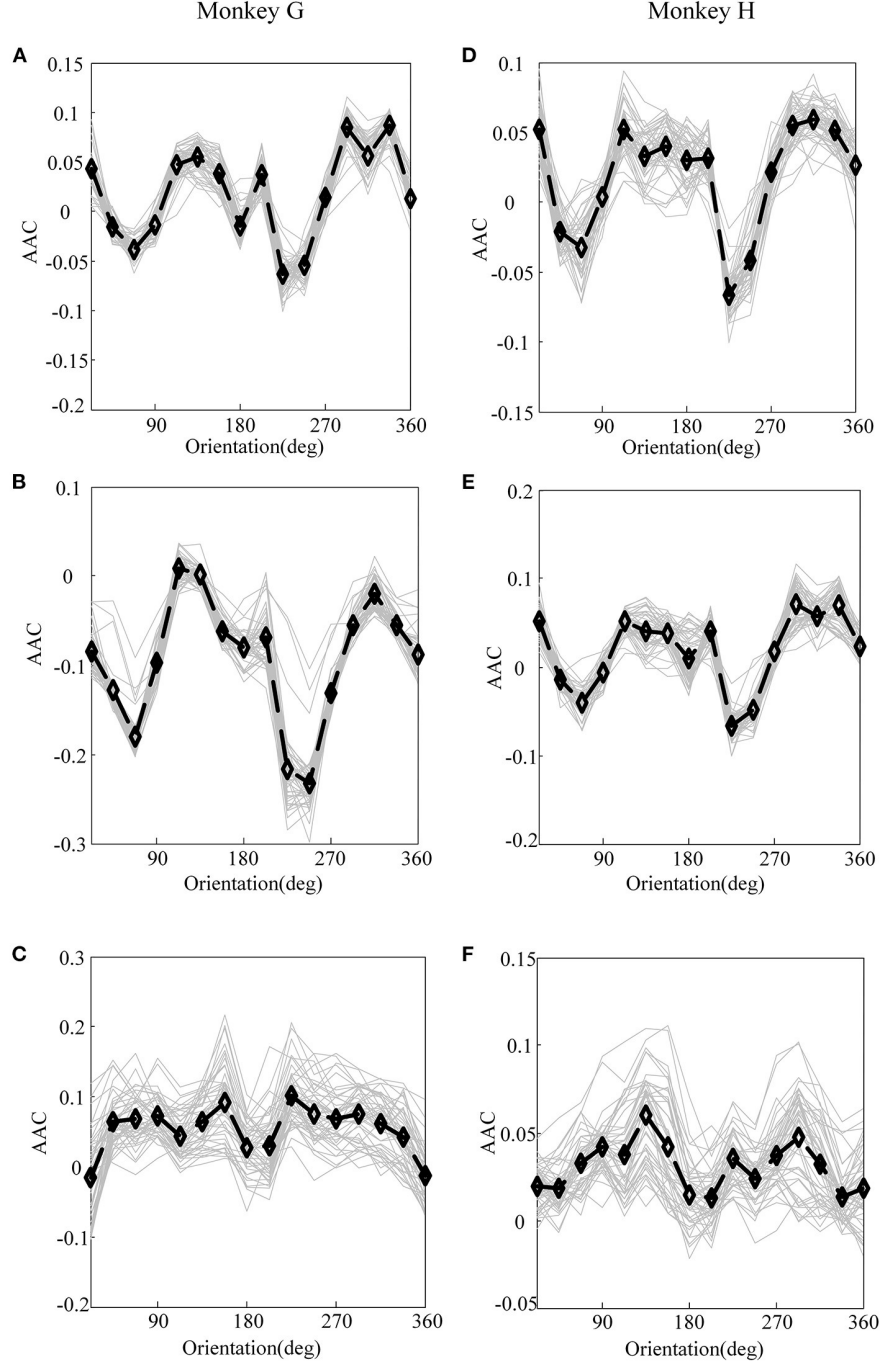
The amplitude–amplitude coupling (AAC) of LFP for monkey G and monkey H. **(A)** The AAC between V1 and V4 of monkey G. **(B)** The AAC in V1 of monkey G. **(C)** The AAC in V4 of monkey G. **(D)** The AAC between V1 and V4 of monkey H. **(E)** The AAC in V1 of monkey H. **(F)** The AAC in V4 of monkey H. In these panels, the gray lines represent the AAC of LFP recorded by individual electrodes, and the black lines plot their means.

First, before constructing the LASSO model, it is necessary to investigate the correlation between the cross-frequency coupling (PAC and AAC of the LFP) and the grating orientation in V1 and V4. Thus, we used the eegfilt.m function in the EEGLAB toolbox (Delorme and Makeig, 2004) to extract neural oscillations in different frequency bands from the raw recordings. Specifically, in the calculation of PAC, the low-frequency oscillations were obtained by using a 0–30-Hz band-pass filter, and the high-frequency oscillations were acquired by utilizing a 30–200-Hz band-pass filter (Esghaei et al., 2015). The diagrammatic sketch for the computing process of PAC is illustrated in Figure 2. Also, we can get the AAC in a similar way. The PAC and AAC between the low-frequency and high-frequency LFP for all the recording electrodes in V1 and V4 are illustrated in Figures 3, 4, respectively. In fact, before performing the CFC analysis, we have removed the trials with no signals. After getting the results, the values outside the range of mean ± 2 standard deviations were considered as outliers and excluded. Clearly, the cross-frequency coupling strengths, including PAC and AAC, exhibit obvious orientation selectivity. It means that they are effective indices for characterizing the orientation selectivity. More concretely, although the PAC and AAC respond to the non-preference-oriented stimulus, and the preferential orientation stimulus are significantly different, the PAC reflects the grating orientation more clearly than the AAC. On the other hand, the values of PAC and AAC in V1 are greater than that in V4, and the values for monkey H are relatively lower than that for monkey G. The main reasons for these differences are listed as follows: One is that there are individual differences between the two monkeys, and consequently, their responses to the drift gratings are not identical. Another one is that the electrodes located in the V4 of Monkey H are probably close to the color-coded region in the experiment.

**Figure 5:**
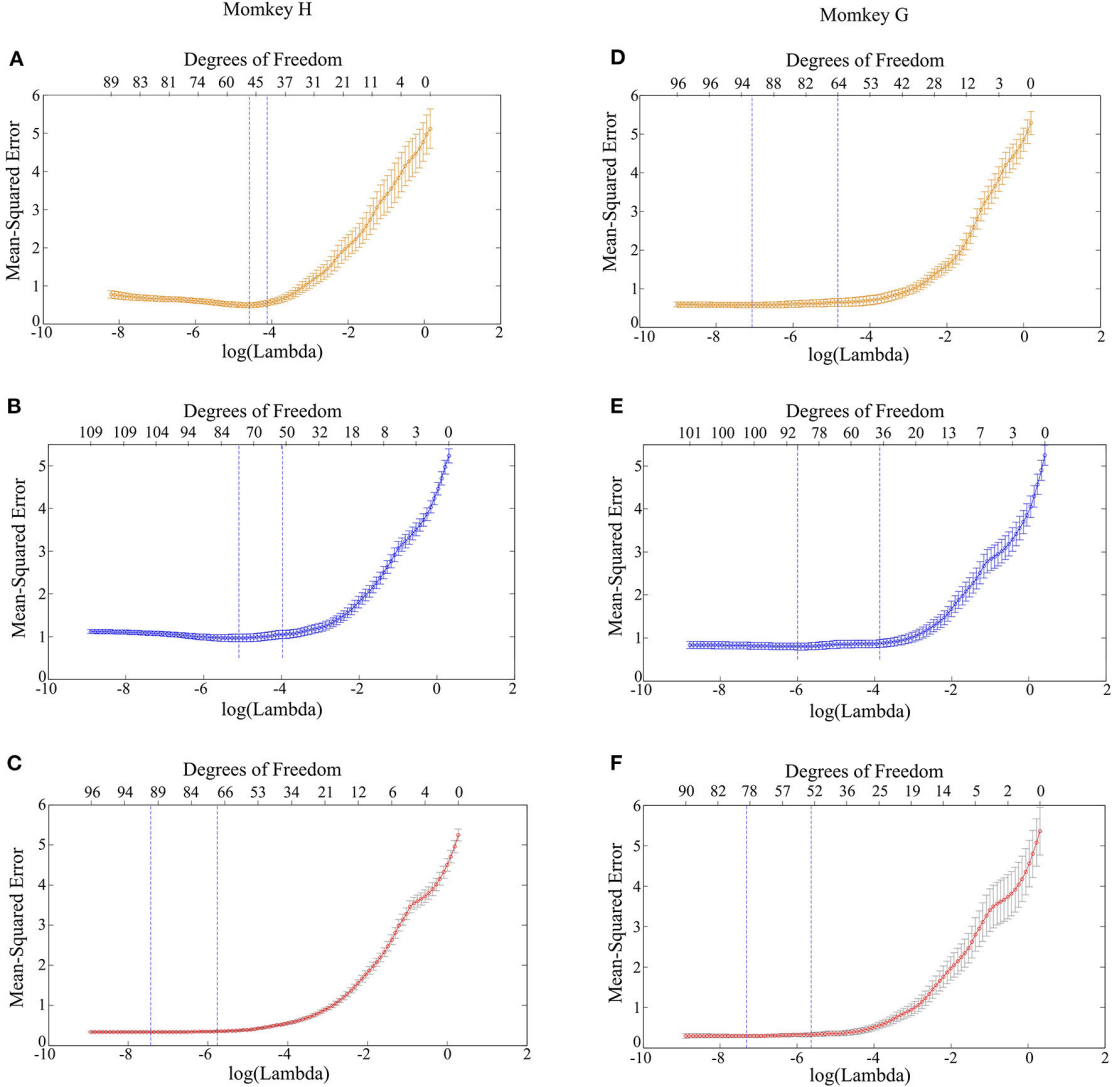
Mean square error of the LASSO model trained with different features. **(A)** PAC for monkey H. **(B)** AAC for monkey H. **(C)** Combined features of PAC and AAC for monkey H. **(D)** PAC for monkey G. **(E)** AAC for monkey G. **(F)** Combined features of PAC and AAC for monkey G.

Next, it is feasible to construct the LASSO model with PAC and AAC in order to predict the grating orientations. The results of cross-validation for Monkey G and H are demonstrated in Figure 5. It can be found that when the model was trained with an individual feature, i.e., PAC or AAC separately, the mean square error of cross-validation is relatively high. While the model was trained with combined features, i.e., PAC and AAC together, the mean square error of cross-validation is small. This means that the LASSO model works more robustly with the two combined features.

Then, we randomly chose four experiments from the cross-validation test as examples, which were denoted as EX1, EX2, EX3, and EX4. The prediction accuracy of the four experiments is shown in Table 1. Obviously, the LASSO model with the input of PAC or AAC separately performs similarly to predict the grating orientation. In fact, the prediction accuracy of the PAC model is ~2% higher than the accuracy of the AAC model. However, the prediction accuracy of the model with the combined features (PAC and AAC) is significantly higher (*p* < 0.05, two-sided sign test). The improvement is nearly 10%, which means that the LASSO model with combined features performs much better than that with the individual features to predict the orientations. To further illustrate the performance of the model with combined features, the accuracies under different orientations and their average values in the four experiments are listed in Table 2. It can be found that the model works well with similar accuracy for all the orientations.

**Table 1:** The prediction accuracy in the four experiments.

**Feature**	**EX1**	**EX2**	**EX3**	**EX4**	**Average**
PAC	64.9%	67.9%	63.8%	62.4%	64.8%
AAC	62.4%	65.7%	61.5%	60.8%	62.6%
PAC+AAC	74.6%	76.0%	73.2%	71.8%	73.9%

**Table 2:** The prediction accuracy for the LASSO model with the two combined features in the four experiments.

**Experiment**	**EX1**	**EX2**	**EX3**	**EX4**
22.5&202.5	72.5%	73.2%	70.6%	68.4%
45&225	80.2%	81.5%	79.4%	78.8%
67.5&247.5	68.6%	69.3%	66.2%	62.2%
90&270	75.7%	77.1%	74.5%	73.2%
112.5&292.5	77.1%	79.5%	76.7%	75.4%
135&315	78.7%	78.9%	77.2%	77.6%
157.5&337.5	72.2%	75.4%	70.8%	68.1%
180&360	71.8%	72.7%	70.1%	70.7%
Average value	74.6%	76.0%	73.2%	71.8%

## Discussion and Conclusion

We recorded the neural data in V1 and V4 of two rhesus monkeys by utilizing two chronically implanted multi-electrode arrays. In the experiment, the monkeys performed a selective visual attention task, where the stimulus was a drifting sinusoidal grating. Then, we extracted the PAC and AAC available in the LFPs and constructed a LASSO model to predict the grating orientation.

According to previous studies about the primate visual cortex, selectivity for the orientation of a visual stimulus is an emergent property of neurons in V1 (Hubel and Wiesel, 1968; Ferster and Miller, 2000; Ringach et al., 2002a). In fact, V4 also seems to be involved in the processing of orientation information. V4 was originally characterized as a color area (Zeki, 1973, 1983). However, subsequent studies also found prominent orientation selectivity among V4 cells (Schein et al., 1982; Mountcastle et al., 1987; Schein and Desimone, 1990; Roe et al., 2012). Therefore, we simultaneously implanted two 6 × 8 multi-electrode arrays in V1 and V4 to perform the data recording. Then, it is feasible to calculate the PAC and AAC in these two areas to characterize the orientation selectivity. Moreover, cognitive functions rely on the coordinated activity of neurons in different brain regions. Specifically, there are interactions in the link between V1 and V4 (van Kerkoerle et al., 2014; Bastos et al., 2015). Thus, we also investigated the PAC and AAC between the LFPs in V1 and V4, which were used as the input of the LASSO model. In fact, most previous studies about the orientation selectivity were based on neuronal firing activities (Ringach et al., 2002b; Scholl et al., 2013; Mazurek et al., 2014). Instead, we used LFP in a larger scale to construct a prediction model in this study, which could effectively predict the grating orientation. We think the principle behind this model is a novel direction for exploring the orientation selectivity. Specifically, LASSO is a regression analysis method, which performs variable selection and regulation to enhance the prediction accuracy. Relating the manner for selection and regulation of CFC in LASSO with the neural information processing in visual cortex is an alternative avenue to reveal the mechanism for generating orientation selectivity. However, there is still a long way to achieve this goal, and we will make more efforts in future studies.

**Figure 6:**
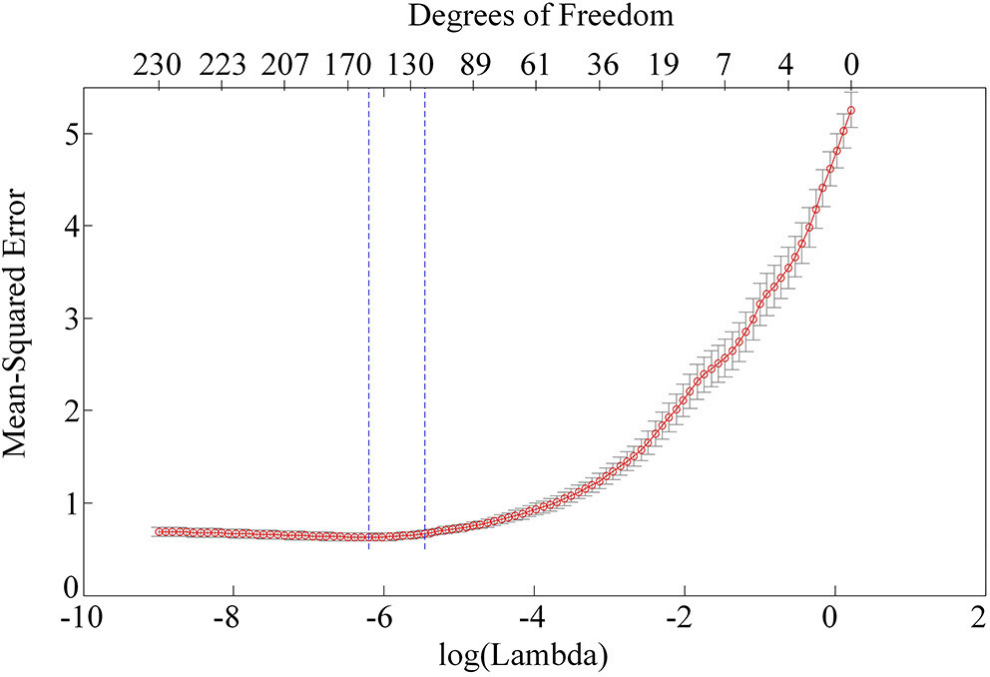
Mean-square error of the LASSO model with three features.

As far as the features for characterizing the orientation selectivity are concerned, the firing spikes of neurons is indeed the most frequently used. However, our results show that the PAC and AAC of the LFPs in V1 and V4 also exhibit diverse preferences to related orientations. While LFP reflect the oscillation of an ensemble of neurons, spikes are the firing activities of an individual neuron (Katzner et al., 2009; Buzsáki et al., 2012). Then, it is necessary to examine the performance of the LASSO model when the firing rate is considered as an additional input feature. To identify the firing spikes, the recorded raw signals were first filtered by using a 300–3,000-Hz band-pass filter. Then, we determined the spiking time with a threshold detection method and extracted the spike waveforms. Finally, an unsupervised method based on wavelets and superparamagnetic clustering were used to classify the spikes (Quiroga et al., 2004). Figure 6 shows the mean square error of the LASSO model with three features, i.e., PAC, AAC, and spike firing rate. Compared with panel (C) in Figure 5, there is no obvious improvement on the result of cross-validation. Also, the predicting accuracy is not significantly improved (*p* > 0.2, two-sided sign test). Here, only the results of monkey H is presented. A similar result for monkey G can be obtained. The reason for this result is that LFP represents the activity of multiple neurons in a local region (Buzsáki et al., 2012), and the CFC extracted from LFP is probably associated with the spikes fired by individual neurons. Therefore, adding the firing rate as an input feature cannot improve the predicting performance. In future studies, we will use additional responses extracted from LFP as inputs of the LASSO model. Hopefully, an optimal combination of the features can be determined, and consequently, the prediction accuracy and robustness of the model will be improved.

In conclusion, a novel method is proposed in this paper to predict the grating orientation, which is based on the LASSO logistic regression model with two combined features of PAC and AAC. Although the average prediction accuracy of the model is 73.9%, i.e., the method cannot predict the orientation perfectly, it is worthwhile to make some efforts to improve the performance of the model. Our results suggest that the LASSO model can effectively predict the grating orientation, which provides an alternative direction for further research to explain the orientation selectivity.

## Data Availability Statement

The datasets generated for this study are available on request to the corresponding author.

## Ethics Statement

The animal study was reviewed and approved by The Institutional Animal Care and Use Committee of Beijing Normal University. Written informed consent was obtained from the owners for the participation of their animals in this study.

## Author Contributions

ZL contributed to the simulation design and manuscript preparation. YD and YX contributed to performing the simulations and analysis. LY contributed to the manuscript preparation. All authors contributed to the article and approved the submitted version.

## Conflict of Interest

The authors declare that the research was conducted in the absence of any commercial or financial relationships that could be construed as a potential conflict of interest.
